# Bioleaching Mercury from Coal with *Aspergillus flavus* M-3

**DOI:** 10.3390/microorganisms11112702

**Published:** 2023-11-03

**Authors:** Wenqing Mao, Juan Mei, Huan He, Cheng Liu, Xiuxiang Tao, Zaixing Huang

**Affiliations:** 1 Key Laboratory of Coal Processing and Efficient Utilization of Ministry of Education, School of Chemical Engineering and Technology, China University of Mining and Technology, Xuzhou 221116, China; ts21040211p31@cumt.edu.cn (W.M.); meijuan808@163.com (J.M.); chengliucumt@163.com (C.L.); taoxx@cumt.edu.cn (X.T.); 2 National Engineering Research Center of Coal Preparation and Purification, China University of Mining and Technology, Xuzhou 221116, China; zhuang@uwyo.edu; 3 Department of Civil and Architectural Engineering, University of Wyoming, Laramie, WY 82071, USA

**Keywords:** bioleaching, bioliquefaction, mercury, fungi, mercury speciation

## Abstract

This study focuses on the utilization of *Aspergillus flavus*(M-3) for the bioleaching mercury from coal, offering an alternative and environmentally to its clean utilization. The fungus was isolated from the soil near a high mercury coal mine in Lao Ying Shan (LYS), Guizhou. Utilizing direct mercury analysis, X-ray diffraction (XRD), and Fourier Transform-Infrared (FT-IR) analysis techniques, the transformation of mercury speciation, mineral components, and organic groups in the coal were analyzed before and after the bioleaching process. The findings of the study illustrated that the fungus M-3 exhibited a remarkable capacity for coal bioliquefaction and mercury leaching from LYS coal. Following a 15-day bioleaching process, a remarkable mercury leaching rate of 83.79% was achieved. Various forms of mercury speciation, including residue, organic matter, sulfide-bound, oxide-bound, exchangeable, and carbonate-bound forms, were released from the coal, with leaching rates ranging from 80.41% to 92.60%. XRD analysis indicated that the M-3 strain facilitated the dissolution of coal pyrite and the degradation of macromolecules, effectively loosening the coal structure. FT-IR analysis of raw and residual coal demonstrated the breakdown of the aromatic ring structure and introduced oxygen-containing functional groups by M-3. Overall, this study highlights the efficacy of bioliquefying coal using *Aspergillus flavus* (M-3) as a method for clean coal utilization while simultaneously bioleaching mercury.

## 1. Introduction

The primary energy source in China continues to heavily rely on coal, and this situation won’t alter in the foreseeable future [1]. However, the mining and utilization of coal pose significant risks to human health and environmental pollution due to the release of hazardous elements. Among them, mercury stands out as a toxic heavy metal element and the sole gaseous trace metal contaminant [2]. Handling elemental mercury is challenging due to its poor solubility in water, high volatility in the vapor phase, poor thermodynamic stability at high temperatures, and long-range atmospheric transportation [3]. Mercury and its compounds accumulate in the environment and organisms, causing severe damage to the human nervous system, tissues, and organs [4]. Recognizing its detrimental effects, the European Union, the World Health Organization, the United Nations Environment Program, and other organizations have labeled mercury as a priority pollutant requiring control measures [5]. According to the 2018 Global Mercury Assessment Report, approximately 2220 tons of anthropogenic mercury emissions were recorded in 2015, with 22.4% originating from coal-related activities [6]. Notably, coal combustion represents the main contributor to the global anthropogenic mercury emissions.

To reduce mercury emissions from coal combustion, several techniques are employed, including pre-combustion, in-combustion, and post-combustion mercury removal [7]. Among these techniques, those utilizing adsorbents during coal washing, burning, and catalytic oxidation have proven to be more efficient in mercury removal [8]. Additionally, bioleaching offers an environmentally friendly approach to the handling of hazardous substances like heavy metals. Previous studies have showcased the potential of direct bioleaching for extracting Co, Ni, chromium, copper, manganese, zinc, and vanadium using various microbial agents [9,10,11,12]. Interestingly, the bioliquefaction of lignite under the action of microorganisms has demonstrated the conversion of coal into high-value chemicals while facilitating the leaching of trace elements into the liquid phase [9]. Furthermore, the speciation of leached metals undergoes variations throughout the leaching process influenced by microbial and environmental factors. Researchers have observed the solubilization of heavy metals and alterations in the speciation distribution of vanadium during bioleaching processes [13,14].

Despite the existing knowledge on microbial bioleaching of various elements from coal, there are limited reports on bacteria-mediated bioleaching of mercury. Considering the relatively high mercury concentration in Chinese coal compared to the global average, particularly in the southwestern region of China, it becomes crucial to develop methods for mercury removal to enable the clean utilization of coal in this area [10,11]. In this study, our primary objective was to screen microbial strains capable of bioleaching mercury from coal obtained from the Lao Ying Shan (LYS) coal mine in Guizhou, China. Additionally, we investigated the speciation transformation of mercury during the bioleaching process facilitated by a selected fungal strain. Through these investigations, we aim to contribute to the knowledge about mercury bioleaching from coal and provide valuable insights for the clean utilization of coal resources.

## 2. Materials and Methods

### 2.1. Coal Samples Preparation

Coal samples used in this study were obtained from the southwest Chinese province of Guizhou, containing high levels of mercury. The raw coal samples were air-dried and then ground into particles ranging from 0.045 mm to 0.125 mm in size for physicochemical analysis and bioleaching experiments. According to the national standard GB/T214-2007 [15], the content of C, H, O, and N was measured using an elemental analyzer (Vario Macro cube, Shanghai, China), and the total sulfur content of coal samples was determined by sulfur sizer (CDS3000, Xuzhou, China). The moisture content was determined by the electric blast dryer (ZHWY-211C, Shanghai, China), and the ash content was determined by the slow ash method using a muffle furnace (CTM-300, Xuzhou, China). The volatile content was determined, and the fixed carbon content in the coal was calculated according to the results of GB/T476-2008 [16]. The composition and relative content of minerals in coal were conducted using an X-ray diffractometer (Bruker D8 Advance, Saarbrucken, Germany), while the functional groups of basic properties of coal samples were determined by an FT-IR spectrometer (Bruker VERTEX 80v, Saarbrucken, Germany).

### 2.2. Screening and Identification of Mercury Leaching Fungus from Coal

Fresh surface soil samples were obtained from the LYS coal mine and mixed with sterile saline. The resulting soil solutions were diluted to a concentration of 10^−2^–10^−5^. Then 0.1 mL of each dilution was uniformly distributed on PDA (HB0233-12, Haibo Bio, Qingdao, China) solid medium, and cultured at a steady 30 °C incubator. Every 2 days, the clone on the plate was observed. The microorganisms in the soil dilutions were sorted into single colonies with a consistent morphology and independent distribution through the plate scribing method. The PDA medium was sprinkled with 0.3 g of coal samples respectively, and the bioliquefaction of coal was observed every 2 d. The formation of black droplets on the plate indicated the strain had the potential to bioliquefy coal, as reported in the literature [12]. The coal samples that produced black droplets were collected from plates and rinsed 3 times with deionized water before being analyzed for mercury concentration using a direct mercury analyzer (DMA-80, Modena, Italy). The mercury leaching rate was determined according to the following formula:

(1) $$P=\left(1-\frac{W_{1}}{W_{0}}\right)\times 100\%$$

where *P* represents the leaching rate of mercury (%), *W*_0_ represents the mercury content of raw coal (g), and *W*_1_ represents the mercury content of residual coal (g) after bioleaching. Finally, 12 fungi were discovered to have the capacity to leach mercury. A strain M-3 with the best mercury leaching rate was selected as the research object and the molecular identification was processed by DNA extraction, and PCR amplification with NS1 and NS6 as primers. The PCR products were sent to Shanghai Biotech for sequencing.

### 2.3. Determination of Physicochemical Properties and Speciation Analysis of Mercury during Bioleaching Coal 

In accordance with our previous work, potato 30 g, glucose 3 g, and deionized water 150 mL were used to prepare the PD liquid medium [17]. For the experiment, 42 bottles of PD liquid medium were sterilized, and 21 bottles of the medium were inoculated with 10% fungal solution, whereas the other 21 bottles were used as control. After inoculation treatment, the mycelium growth was monitored, and 3 g of treated coal sample (1% surfactant fatty alcohol-polyoxyethylene ether AE07) was added to the 21 bottles of the blank medium group and three parallel replicate tests were conducted. The culture solutions of the control and microbiological groups were sampled every three days for 21 days while coal was being bioleached, and a new PD liquid medium was added before and after each sampling. Three sets of parallel samples were measured each time, and the pH and ORP values of the culture solution were determined using a digital pH/ion meter (PHS-25, Leici, Shanghai, China), and the Ec values of the culture solution were determined using a conductivity meter (DDS-11A, Leici, Shanghai, China). All the results were presented with the average value. Every 3 days, the residual coals were also extracted using the chemical extraction method [18]. We used 1 mol/L MgCl_2_, 1 mol/L NaAC/HOAC, 0.04 mol/L NH_2_OH·HCl, HNO_3_, HF, and HClO_4_ solutions to extract mercury exchangeable state, carbonate bound state, oxide bound state, organic matter, and sulfide bound state and residue state in coal step by step, respectively. The Hg content in each step was measured separately with a direct mercury analyzer (DMA-80, Modena, Italy) to obtain the content of different Hg speciation. 

### 2.4. Analysis of Mineral Composition and Organic Functional Groups of Coal before and after Bioleaching

After 21 days of bioleaching, the culture solution was filtered (1 mm pore size) and the collected coal samples were transferred into the plate and placed in a 60 °C oven and frozen drier for sample preparation, respectively. The peak position (θ), area (A), and full width at half maximum (β) parameters were retrieved from the XRD spectrum using the Jade 6.5 software. Then, interlayer spacing (d002), lateral size (La), stacking height (Lc), and stacking layers(N) were characterized with XRD and a semi-quantitative analysis [19]. The FTIR peak fitting analysis was performed using Origin9.1 software (Origin Lab Corporation, Northampton, MA, USA) to provide detailed information on the organic functional group of coal. In order to analyze the LYS raw coal and residual coals, the relevant basic parameters of the coal were determined, including four structural parameters (fa, I, DOC, and “C”) using spectra data [13]. The measure of aromaticity (I), which can be used to describe the relative abundance of aromatic and aliphatic groups, was determined using Equation (5). The degree of condensation of aromatic rings (DOC) was determined using Equation (6), and the oxygenated structure of the coal was determined by “C” which calculates the change in the ratio of C=O group to C=C group, as shown in Equation (7).

(2) $$\frac{H_{\mathrm{al}}}{H}=\frac{H_{\mathrm{al}}}{H_{\mathrm{al}}+H_{\mathrm{ar}}}=\frac{A_{3000-2800}}{A_{3000-2800}+A_{900-700}}$$

(3) $$\frac{C_{\mathrm{al}}}{C}=(\frac{H_{\mathrm{al}}}{H}\times \frac{H}{C})/\frac{H_{\mathrm{al}}}{C_{\mathrm{al}}}$$

(4) $$f_{a}=1-\frac{C_{\mathrm{al}}}{C}$$

(5) $$I=\frac{A_{900-700}}{A_{3000-2800}}$$

(6) $$\mathrm{DOC}=\frac{A_{900-700}}{A_{1600}}$$

(7) $$“C”=\frac{A_{1800-1650}}{A_{1800-1650}+A_{1600}}$$

## 3. Results and Discussion

### 3.1. Isolation and Identification of Fungal Strain with Coal’s Bioliquefaction and Hg Bioleaching Ability

**Figure 1:**
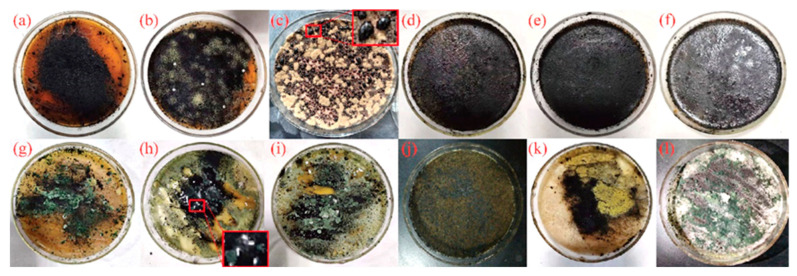
Bioliquefaction test of raw coal by the isolated strains. (**a**): M-1, (**b**): M-2, (**c**): M-3 (10×), (**d**): M-4, (**e**): M-5, (**f**): M-6, (**g**): HN-1, (**h**): HN-2 (10×), (**i**): HN-3, (**j**): HN-4, (**k**): HN-5, (**l**): HN-6.

To isolate fungi with the ability to liquefy coal and leach Hg, enrichment and isolation procedures were carried out on soil samples collected from the LYS coal mine. A total of 12 fungal strains were obtained from the soil around the LYS coal mine, and their bioliquefaction test on coal is shown in Figure 1. As can be seen, there were some small black droplets on the surface of the solid medium inoculated with HN-2 and M-3, while the other solid media did not show any liquefaction of coal. Similarly, black droplets were found on the surface of the medium when the fungus was used to liquefy low-grade Thar coal [14]. Shi et al. [12] studied black droplets were formed on the surface of sand maltose agar (SMA) medium through *Hypocrea lixii* AH solubilized coal, and that the water content in the droplets originated from the medium and biological oxidation rather than from the coal. 

**Figure 2:**
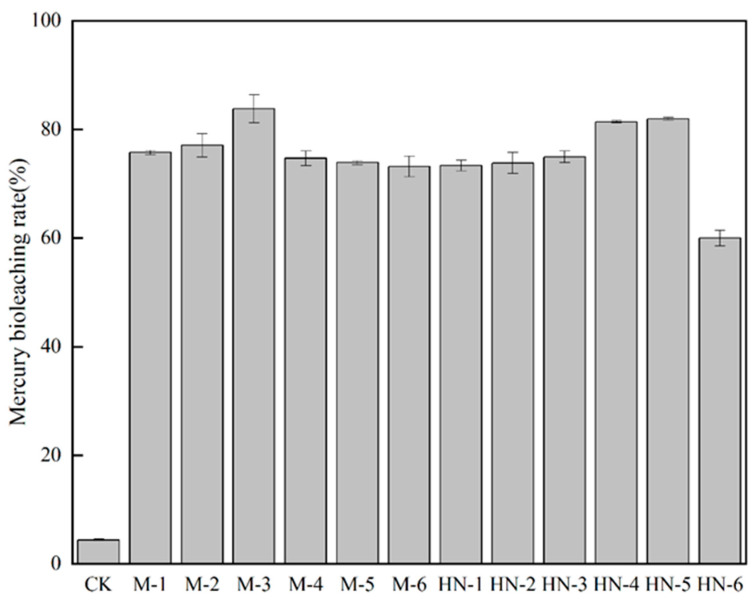
Bioleaching rate of mercury by Isolated strains on solid media. Note: Error bars indicate standard deviation, the same below.

Figure 2 shows that M-3 has the highest leaching rate (up to 83.57%), which is consistent with the effect of coal bioliquefaction. Based on the bioliquefaction and bioleaching results, the strain M-3 was chosen for the following tests. The molecular sequencing results of M-3 were blasted, and the results showed that M-3 had the highest similarity with *Aspergillus flavus* HQ393872.1, so the M-3 fungus was assigned as *Aspergillus flavus* M-3.

### 3.2. Analysis of pH, ORP, Ec, and Total Hg Content during Bioleaching of Mercury from Coal by the Fungal Strain M-3

**Figure 3:**
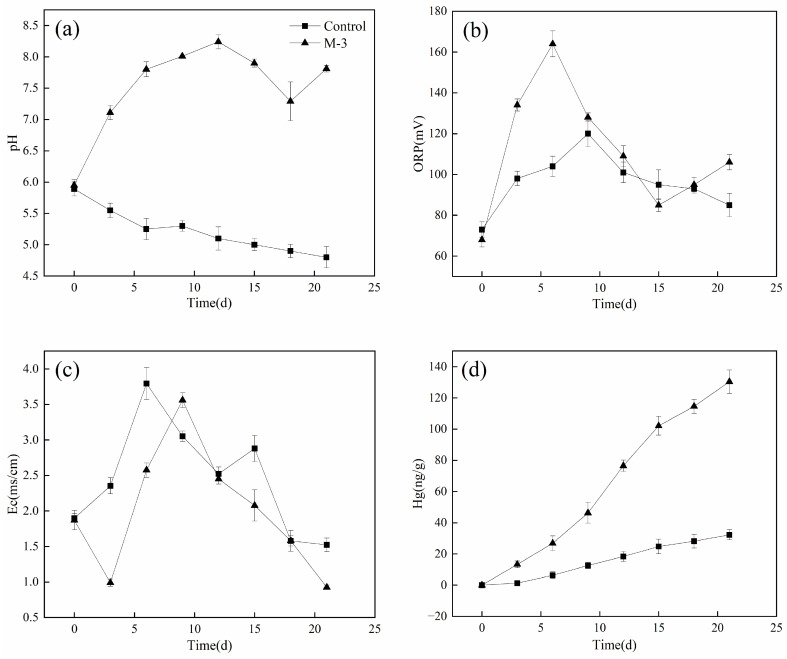
Changes in pH (**a**), ORP (**b**), Ec (**c**), and Hg (**d**) of the culture solution during coal bioliquefaction.

The change curves of pH, ORP (Oxidation Reduction Potential), Ec, and the total mercury content in the leaching solution during the treatment of LYS coal by the microbial group and control group are shown in Figure 3. Figure 3a illustrates that the initial pH of the PDA liquid medium is weakly acidic, and the pH of the liquefaction system of the microbial group continues to increase during the first 12 d, peaking at 8.24 on 12 d and then decreasing gradually. This is consistent with the results of previous studies [20], that have demonstrated fungi’s ability to dissolve lignite employing auto-secreted alkaline products [12,21]. Hence, we presumed that the increase in pH of the liquefaction system was attributed to the extracellular alkaline substances secreted by M-3. Additionally, based on prior reports [22], the pH tends to decrease after 12 d probably because the leaching reached saturation and metabolic waste gradually accumulated. After the 12 d bioleaching, M-3 entered the decay stage, resulting in a decrease in secreted alkaline compounds. In contrast, the control group pH maintained weakly acidic, which slowly decreased, possibly due to the dissolution of soluble humic acid from coal.

Figure 3b depicts the ORP trend of in the culture solution. In general, the ORP values did not change obviously in the microbial and control groups, although the former generally exhibited higher values. Within the first 6 d, both the control and microbial groups experienced an increase in ORP values, with the microbial group reaching the highest value of 164 mV, and then continued to decrease, finally recovering slightly after 15 d. The ORP of the control group peaked at 120 mV on the 9th day, and then steadily decreased. Generally, ORP is typically considered a critical factor in controlling the leaching rate in certain bioleaching systems [23,24], with higher values indicating enhanced microbial efficiency [24,25]. In the microbial group, the trend of ORP correlated with the mercury leaching rate, increasing until 6 days and declining thereafter, likely due to decreased microbial activity at that stage.

As shown in Figure 3c, the Ec values presented minimal changes following fungal inoculation. The microbial group showed a decreasing trend from 0–3 d, followed by an increase, reaching a maximum of 3.56 mS/cm on the 9th day, and subsequently declining. Conversely, the Ec of the control group started to rise, reaching a maximum of 3.795 mS/cm on the 6th d and then decreased, with an overall conductivity higher than the microbial group. In Figure 3d, the total mercury of both the microbial and control groups continued to increase during the bioleaching process, with the microbial group exhibiting higher total mercury content compared to the control group, indicating improved leaching of mercury by the fungi. With the leaching of mercury, the pH displayed an increasing trend, likely influenced by the presence of alkaline substances during the coal bioliquefaction process. 

### 3.3. Mercury Speciation Analysis in Coal during Bioleaching by the Fungal M-3

**Figure 4:**
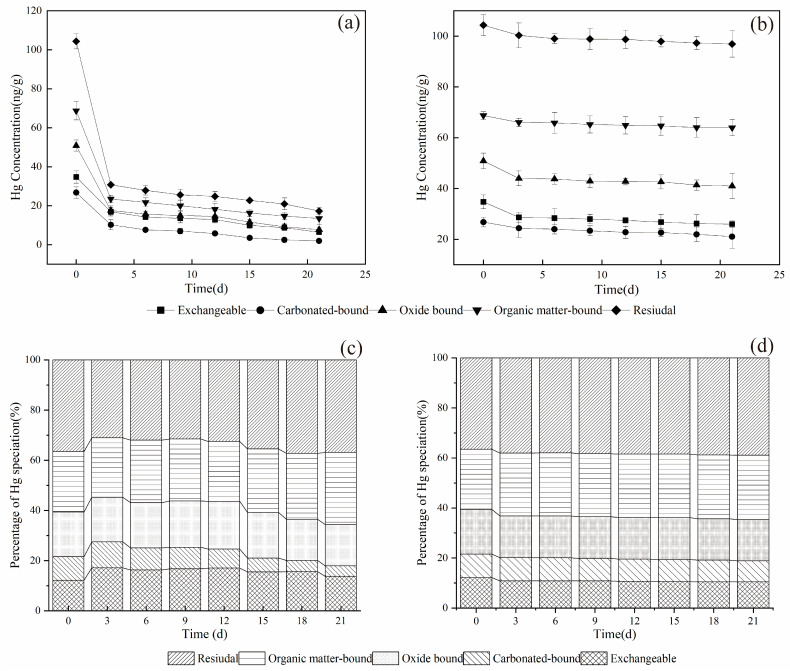
Mercury speciation in coal samples of microbial group (**a**,**c**) and control group (**b**,**d**).

Figure 4 shows the total content and percentage changes of the different mercury speciation during the coal bioleaching. Elemental mercury can exist in five speciations: exchangeable, carbonate-bound, oxide-bound, organic matter and sulfide-bound, and residues [26,27]. Among these, the exchangeable, carbonate-bound, iron/manganese oxide-bound are considered more mobile, hazardous, and bioavailable than the organic matter/sulfide and residue speciation [28]. Changes in major cationic composition or lowering of pH may cause their release due to ionic exchange and/or carbonate dissolution [28]. Under decreasing pH conditions, Fe/Mn oxides, substances, and sulfides tend to be relatively stable. However, Fe/Mn oxides exhibit higher biological effectiveness under reducing conditions, while substances and sulfides exhibit more activity under oxidizing conditions. Metals in the residue form are generally stable and not easily released under normal conditions [29]. 

From Figure 4a,b, it is evident that the mercury content in various speciation significantly decreased after the bioleaching of raw coal. After 21 days of fungal treatment, the mercury content was 46.910 ng/g (sum value), while in the raw coal was 285.495 ng/g. This indicates that M-3 can reduce elemental mercury content in high mercury coal by 83.57%, resulting in different speciation distributions. The distribution trend of mercury during fungal treatment followed the order: of residue form > organic matter and sulfide bound form > oxide bound form > exchangeable form > carbonate bound form. 

In Figure 4c,d, it can be observed that after microbial treatment, the percentage of exchangeable mercury in the coal initially increased and then decreased over time. It accounted for 12.17% of the raw coal, increased to 17.13% at 3 d, and gradually decreased to 13.71% at 21 d. This phenomenon may be attributed to M-3 acting on the carbonate-bound form initially, breaking its chemical bonds and thus partially converting it to the exchangeable form [30,31]. Correlation analysis of mercury speciation with pH, ORP, Ec, and Hg was conducted (Figure S1), revealing a significant correlation between the proportion of carbonate-bound form and total mercury content. The reduction of the carbonate binding state ratio with leaching further supported this observation. The percentage of the oxidatively bound form of mercury did not show significant changes before and after microbial treatment. The oxide-bound form is generally unstable under reducing conditions. In this experiment, the oxidation potential of the culture solution presented an oxidizing environment, so overall, the ORP value has little effect on mercury speciation change. Similar findings were reported by Zhang et al. [32], concluding that the oxide-bound form had a limited effect on the distribution of most heavy metals in the active form. The percentage of organic matter and sulfide-bound form of mercury in the coal from the microbial group tended to increase over time. This increase in the microbial group may also be attributed to the faster leaching rate of this form compared to other forms into the organic-bound form [30,31]. Zhang et al. [33] proposed that an increase in pH favors the accumulation of organic bound forms of heavy metal ions, and there is an overall increasing trend in pH during leaching. These results suggested that bioleaching strongly influenced the percentage distribution of mercury and affected the transformation of mercury speciation during the leaching process [27]. The increase in the proportion of organic bound forms in this study further supports these findings. In addition, in the control group, there was a significant correlation between the pH of the culture medium and various forms of mercury without the action of microorganisms (Figure S1), indicating the crucial role of pH in mercury leaching.

### 3.4. Proximate, Ultimate, and XRD Analysis before and after Bioleaching Coal by the Fungal Strain M-3

**Figure 5:**
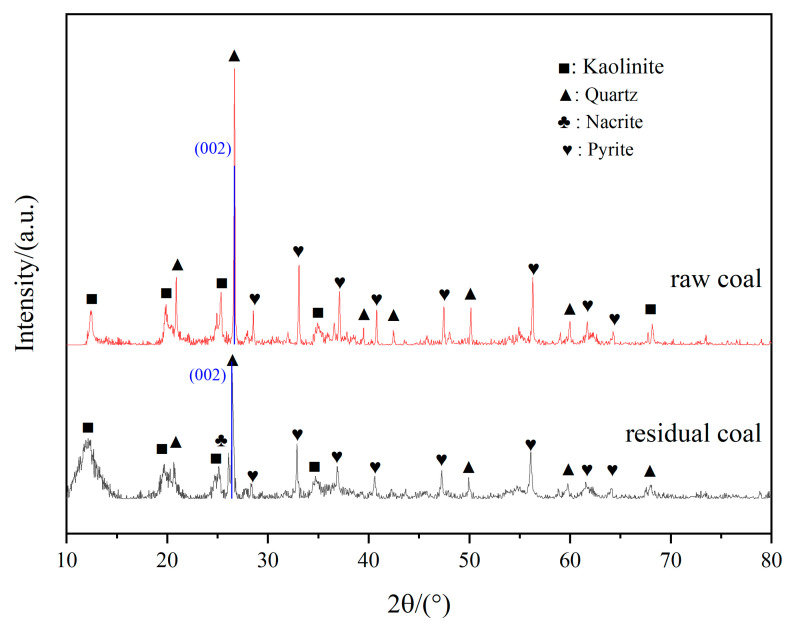
XRD spectra of raw coal and residual coal before and after bioleaching.

The proximate and ultimate of coal before and after bioleaching by the fungal strain M-3 are presented in Table 1 and the XRD analyses of coal are presented in Figure 5. The raw coal sample exhibited high ash content (53.01%), moderate volatile matter (21.02%), and fixed carbon (23.93%) as well as low sulfur content (1.13%), classifying it as high ash and low sulfur coal. The mercury content in the coal was 300.02 ng/g, indicating it as as high mercury coal. After treatment with M-3, the moisture, ash and volatile fraction increased while the fixed carbon decreased compared to the raw coal. The volatile matter primarily consists of oxygen-containing functional groups and small molecules resulting from the breakage of aliphatic side chains [34]. The increase in volatile fraction indicated the addition of oxygen-containing functional groups as well as aliphatic side chain breakage. Following M-3 treatment, the nitrogen, oxygen and hydrogen content increased by 0.84%, 1.28%, and 0.52%, respectively, while the carbon and sulfur content decreased by 2.04% and 0.85%, respectively [35]. The increase in oxygen content indicated that the oxygen-containing functional groups increased, which was consistent with the results of previous studies [9]. These results suggested that strain M-3 could dissolve more carbon and sulfur from raw coal, releasing them into the soluble fraction while retaining most of the ash in the residue.

Figure 5 presents the XRD results of the raw coal and residual coal. Both samples mainly consist of kaolinite, quartz, nacrite, and pyrite. Compared to the raw coal, the residual coal remained unchanged in mineral composition, but the relative content (partially reflected by the intensity of peaks) of certain minerals changed. Pyrite, a common metallic sulfide mineral containing various heavy metals [36], could be effectively reduced through the leaching action of M-3.

The X-ray diffraction (XRD) peaks were analyzed using Origin 9.1 software, and the corresponding structural parameters of the coal micro-molecular structure are presented in Table 2. Previous studies have indicated that the observed overlapping peaks at 26.48–26.68 cannot be exclusively attributed to quartz; they also correspond to the macromolecule structure of coal, specifically referring to graphite [19,37]. The coal consists of multiple aromatic ring layers arranged in different degrees of parallelism. The 002 peak in Figure 5 can be attributed to the spacing between these aromatic ring layers. To accurately determine the location of the 002 peaks, we excluded the contribution from mineral peaks in our analysis. Furthermore, as the microorganism under investigation does not interact with quartz, any changes observed in the overlapping peaks can be attributed to alterations in the coal (referring to graphite) structure. The interlayer spacing, denoted as d_002_, was found to be larger in the microbially degraded residual coal (0.34 nm) compared to the interlayer spacing in the raw coal (0.33 nm). This indicates an increase in interlayer spacing following bioleaching. These findings align with previous studies [34] in which the gradual opening of aromatic rings and the introduction of oxygen-containing groups at the break sites resulted in the formation of three-dimensional structures within the coal matrix. The bond spacing of these three-dimensional structures was larger than that of the aromatic nucleus, leading to an increase in the interlayer distance, specifically d_002_ [38]. The later size (*L_a_*), stacking height (*L_c_*), and number (N) of the aromatic layer decreased after M-3 treatment. During the leaching process, the extent of aromatic nucleus condensation decreased [37]. The degradation products, consisting of a great number of small molecules, filled the coal micropores, resulting in a decrease in spacing between aromatic layers and a decrease in the size of the aromatic layers. M-3 also utilized some of the small molecules during degradation, leading to a gradual decrease in the stacking height and number of aromatic layers, ultimately causing the coal structure to become less consolidated. These results further confirm the leaching of mercury from coal.

### 3.5. FT-IR Analysis before and after Bioleaching Coal by Fungal Strain M-3

**Figure 6:**
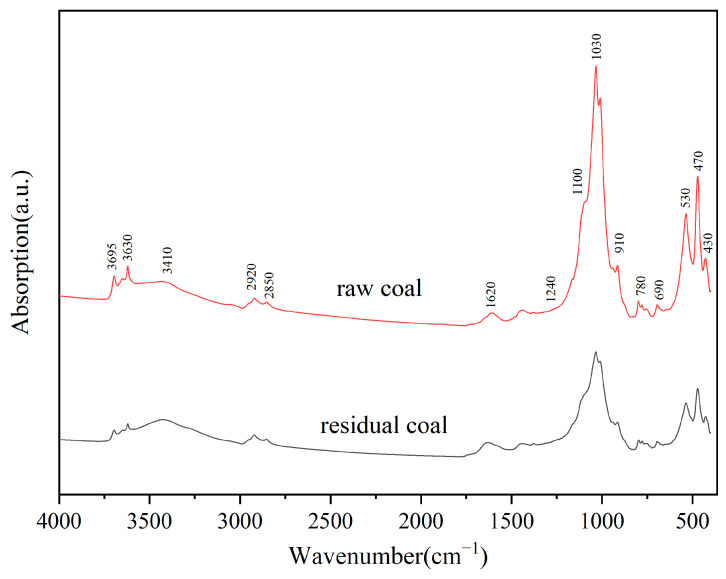
FT-IR spectra of raw and residual coal before and after bioleaching.

The FT-IR spectra of coal samples before and after bioleaching are shown in Figure 6, and correlation fitting analysis is shown in Figure S2. Table 3 provides the positions, areas, and corresponding functional groups of the fitted peaks of the raw and residual coal. 

The spectra exhibited high-intensity sharp peaks around 3695 cm^−1^ and 3630 cm^−1^, assigned to the stretching vibrations of free −OH. These vibrations are crucial for an important basis for determining the presence of functional groups such as alcohols, phenols, and organic acids [12]. The broad and scattered spectral peak around 3410 cm^−1^ in the coal represents the stretching vibration of O-H, hydrogen bonding, and N-H of alcohols, phenols, and carboxylic acids [39]. An increased area of this peak in the residual coal indicated that the microorganisms cleaved various structures, resulting in a hydrogen bonding effect. Peaks at 2920 cm^−1^ and 2850 cm^−1^ are assigned to the asymmetric stretching vibrations of -CH_2_ and −CH groups, respectively [40]. The peaks around 1620 cm^−1^ are -O-substituted C=C stretching vibrations, and hydrogen bond resonance formed by C=O and −OH groups. The intensities of these fitted peaks were stronger in the residual coal, indicating an increase in oxygen-containing functional groups resulting from the action of M-3 on the coal. This finding is consistent with the elemental and XRD analyses. The sharp peaks near 1030 cm^−1^ are assigned to minerals such as quartz, kaolinite, illite, and montmorillonite. The weaker peak intensity of the residual coal indicated the dissolution of minerals. 

According to previous studies, the FT-IR spectra can be divided into four absorption band spectra for segmentation, hydroxyl absorption band (3700~3000 cm^−1^), fatty structure absorption band (3000~2800 cm^−1^), oxygenated functional group absorption band (1800~1000 cm^−1^), and aromatic structure absorption band (900~700 cm^−1^) [41]. The segmentation and fitting of the spectra of the four absorption bands are shown in Figure S2.

The fitted peaks of the residual coal were lower than those of the raw coal, indicating the dissolution of the minerals. The peaks at 780 cm^−1^ and 800 cm^−1^ in the curve-fitted spectra of 900–700 cm^−1^ range are the outward stretching vibrations of the aromatic ring C-H. The absorption peaks from 770 cm^−1^ to 735 cm^−1^ represented 1,2-disubstituted benzene. The positions of the fitted peaks for the raw coal and the residual coal were approximately the same.

We utilized Origin9.1 software to process the measured FT-IR spectra and quantify the functional groups of the coal samples before and after bioleaching. This allowed us to calculate the structural parameters of the coal samples. The main absorption peak parameters are shown in Table 4 and the structural parameters are shown in Table 5. 

Aromatic structure parameters and oxygenated structure parameters of the coal samples can be calculated according to Equations (1)–(6). Compared to the raw coal, the *f_al_* value of the residual coal decreased by 26.90% and the I value decreased by 68.71% (assuming only aromatic and aliphatic carbon atoms were present in the coal sample). The *f_al_* denotes the apparent aromaticity of the coal and I denote the relative abundance of aromatics and aliphatic groups. These findings indicated that M-3 effectively degraded the aromatic rings of the coal, which was consistent with the XRD results. Previous studies by Majeke et al. [42] showed that microorganisms could open aromatic rings by secreting Lip, which catalyzed the cleavage of alkyl side chains and the opening of aromatic rings in lignin-containing compounds. The decrease in DOC in the residual coal by 74.3% further indicated a decrease in aromatic ring polymerization in the presence of M-3, supporting the conclusions of previous studies [12]. The “C” mainly describes the change in C=O. The main forms of C=O groups are ketones, aldehydes, carboxy groups, and esters [43]. Compared with the raw coal, the “C” value of the M-3 treated coal increased by 63.28%, indicating the coal’s oxidation with M-3 by introducing the C=O structure into the structure of the coal [44].

## 4. Conclusions

In the present work, we focused on investigating the effects of bioleaching on the speciation transformation of mercury and the physical and chemical characteristics of high mercury coal from the Guizhou area, using the fungus M-3 with good coal solubilization capability as the research subject. Our findings revealed the isolated fungus M-3, a genus of *Aspergillus*, with 99.9% homology with *Aspergillus flavus* HQ393872.1, could bioleach 83.57% mercury from coal during the bioliquefaction process. Furthermore, the pH of the system tended to increase, presumably due to alkaline substances produced by M-3 during the bioliquefaction. The bioleaching of M-3 mercury could influence the speciation transformation of mercury followed by residue state, organic matter, sulfide bound state, oxide bound state, exchangeable state, and carbonate bound state. The M-3 effectively degrades the aromatic ring structure through introducing C=O groups such as ketones, aldehydes, carboxyl groups, and esters. It oxidized coal while also dissolving some pyrite in the coal and degraded macromolecules to loosen the coal’s structure. Our findings indicate that M-3 could be utilized as a potential bioleaching agent for improving the solubilization and bioremediation of high-mercury coal.

## Figures and Tables

**Table 1:** Proximate and ultimate analyses of coal before and after bioleaching with fungal strain M-3 (wt%).

Coal Sample	*M*_ad_ (%)	*A*_ad_ (%)	*V*_daf_ (%)	FC_daf_ (%)	C_daf_ (%)	H_daf_ (%)	O_daf_ (%)	N_daf_ (%)	S_daf_ (%)
Raw	2.04	53.01	21.02	23.93	71.82	5.93	8.69	1.94	1.13
Residual	2.79	58.50	22.83	17.33	69.78	6.45	9.97	2.78	1.98

M (moisture), A (ash), V (volatiles) FC (fixed carbon) ad (air-dried basis), and daf (dry and ash-free basis).

**Table 2:** Structural parameters derived from the fitted XRD spectra.

Coal Sample	2θ_(002)_/(°)	FWHM_(002)_/(°)	2θ_(γ)_/(°)	FWHM_(γ)_/(°)	*d*_002_ (nm)	*L_c_* (nm)	*L_a_* (nm)	N
Raw	26.68	0.11	26.46	0.89	0.33	1.27	3.50	4.81
Residual	26.48	0.22	26.11	0.15	0.34	0.63	1.13	2.89

**Table 3:** Fitted peaks of functional groups of the raw coal and residual coal.

Peak	Peak Center (cm^−1^)		Peak Area		Functional Groups
	Raw Coal	Residual Coal	Raw Coal	Residual Coal	
P_1_	3695.92	3695.81	0.05	0.02	O-H stretching
P_2_	3630.93	3632.35	0.21	0.06	
P_3_	3433.60	3411.92	1.12	1.22	Hydrogen bond
P_4_	2919.50	2922.07	0.09	0.07	CH_2_ asymmetrical stretching
P_5_	2851.54	2854.30	0.04	0.05	CH asymmetrical stretching
P_6_	1614.80	1623.49	0.10	0.21	C=C stretching
P_7_	1228.46	1263.54	0.93	0.00	C-O stretching
P_8_	1094.24	1191.38	1.43	1.04	
P_9_	1021.49	1035.58	2.30	1.62	Si-O-Si or Si-O-C stretching
P_10_	927.31	908.88	0.47	0.08	C-O stretching
P_11_	781.12	777.85	0.08	0.04	C-H out-of-plane bending
P_12_	691.77	691.34	0.03	0.01	
P_13_	536.02	535.65	0.71	0.33	C-X stretching
P_14_	471.01	469.21	0.48	0.21	S-S stretching
P_15_	429.77	427.61	0.16	0.08	

**Table 4:** Parameters of main adsorption peaks.

Coal Sample	A_900–700_	A_1600_	A_1800–1650_	A_3000–2800_	H/C	H_al_/C_al_
Raw	0.1153	0.0931	0.0084	0.1263	0.9908	1.8000
Residual	0.0345	0.1083	0.0317	0.1207	1.1092	1.8000

**Table 5:** Structural parameters of coal samples.

Coal Sample	H_al_/H	C_al_/C	f_al_	I	DOC	“C”
Raw	0.5227	0.2877	0.7123	0.9132	1.2395	0.0831
Residual	0.7778	0.4793	0.5207	0.2857	0.3186	0.2263

## Data Availability

Data are contained within the article and Supplementary Materials.

## Supplementary Materials

The following supporting information can be downloaded at: https://www.mdpi.com/article/10.3390/microorganisms11112702/s1. Figure S1. Correlation heat map; (a) microbial group, (b)control group. Figure S2. Peak fit spectra of coal before and after bioleaching.
