# Supplementary material for: Bioleaching Mercury from Coal with Aspergillus flavus M-3
Source: Microorganisms. 2023 Nov 3;11(11):2702. doi: 10.3390/microorganisms11112702 (PMC10672889; doi:10.3390/microorganisms11112702)
Supplement: Supplementary file 1 [file microorganisms-11-02702-s001.zip › microorganisms-2678793-supplementary.pdf]

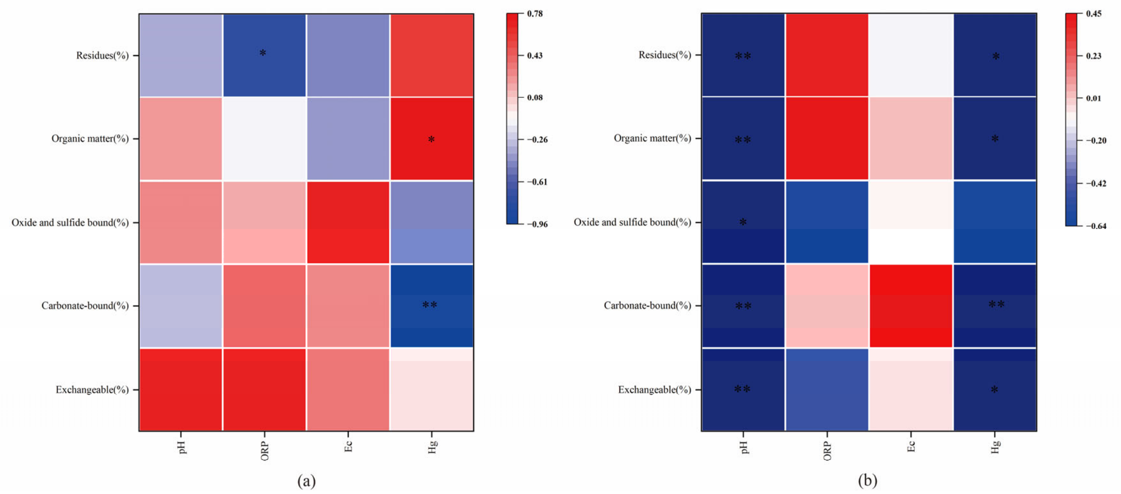

Figure S1. Correlation heat map; (a) microbial group, (b)control group.

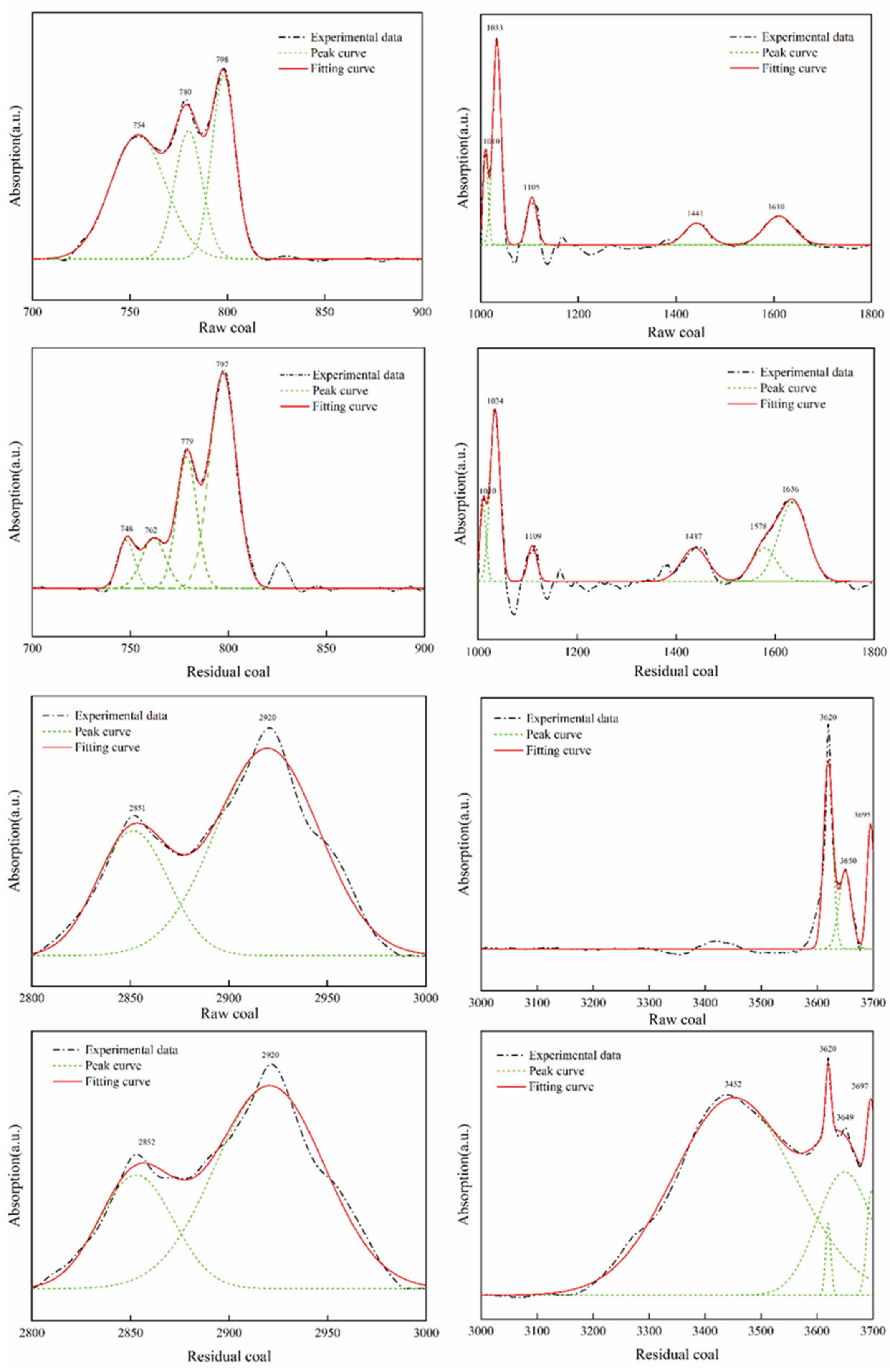

Figure S2. Peak fit spectra of coal before and after bioleaching.
